# Case report: pulmonary artery perforation during transseptal puncture for left atrial appendage closure requires emergency cardiac operation

**DOI:** 10.3389/fcvm.2023.1218582

**Published:** 2023-10-10

**Authors:** Yue Wang, Beibei Song, Bing Liu, Hui Zhang, Chenglong Bi, Wenhao Liu, Gang Ma, Bo Li

**Affiliations:** ^1^ Department of Clinical Medicine, Shandong First Medical University (Shandong Academy of Medical Sciences), Jinan, China; ^2^ Department of Cardiology, Zibo Central Hospital, Zibo, China; ^3^ Department of Cardiovascular Surgery, Zibo Central Hospital, Zibo, China; ^4^ Zibo Central Hospital, Binzhou Medical University, Zibo, China

**Keywords:** left atrial appendage closure, transseptal puncture, pulmonary artery perforation, cardiac tamponade, case report

## Abstract

Patients with atrial fibrillation who take a high bleeding risk and are not candidates for oral anticoagulation therapy are increasingly being referred for left atrial appendage closure (LAAC) as an alternative method of stroke prevention. However, certain manipulations performed during the LAAC procedure, such as transseptal puncture (TSP), may potentially result in vessel injury and lead to cardiac tamponade or even fatality. Clinical significance and management strategies associated with these complications remain controversial. A 74-year-old female patient with atrial fibrillation was referred for left atrial appendage occlusion. During the puncture of the atrial septum, the catheter sheath inadvertently exited through the roof of the right atrium and continued to advance, resulting in pulmonary artery perforation. The patient underwent immediate pericardiocentesis and drainage, followed by surgical exploration for suturing the tear in the pulmonary artery and ligation of the left atrial appendage. This represents the first reported case of a pulmonary artery perforation occurring during a transseptal puncture procedure for left atrial appendage closure. The case exemplifies the feasibility of emergency cardiac surgery as a therapeutic intervention.

## Introduction

In recent years, advancements in electrophysiology and structural cardiac interventions have propelled the growing demand for transseptal procedures, primarily encompassing radiofrequency ablation, left atrial appendage closure, and mitral valve repair (1, 2). Left atrial appendage closure is currently an effective treatment for preventing ischemic stroke in patients with non-valvular atrial fibrillation and a high risk of bleeding and embolism, particularly those who cannot take oral anticoagulants due to contraindications or have a CHADS2 score of 2 or higher (3, 4). The manipulation during LAAC, however, has been associated with potentially serious complications including pericardial effusion, air embolization, device embolization, vascular lesions near the heart, ischemic stroke, and even death (5, 6). Physicians must possess a thorough comprehension of relevant complications and adeptly master their corresponding countermeasures. We present a novel case of inadvertent pulmonary artery perforation during transseptal puncture in the context of proposed left atrial appendage occlusion, which was subsequently resolved through surgical suturing of the ruptured vessel and ligation of the left atrial appendage.

## Case presentation

A 74-year-old female, was diagnosed with paroxysmal atrial fibrillation. Past medical history included coronary atherosclerotic heart disease, thrombosis in the left upper limb artery, heart failure, hypertension, ischemic cerebrovascular disease, and surgically treated endometrial cancer. Following coronary stent implantation and left upper extremity artery thrombectomy procedures, the patient received regular treatment with standard-dose oral Aspirin, Clopidogrel sulfate, Pitavastine, Sacubitril-valsartan, Nifedipine, and Metoprolol. The patient presented with a CHA2DS2-VASC score of 7 and a HAS-BLED score of 3, clearly indicating the necessity for left atrial appendage closure. Considering the potential risks associated with long-term anticoagulation therapy, percutaneous LAAC is performed for secondary stroke prevention.

**Figure 1:**
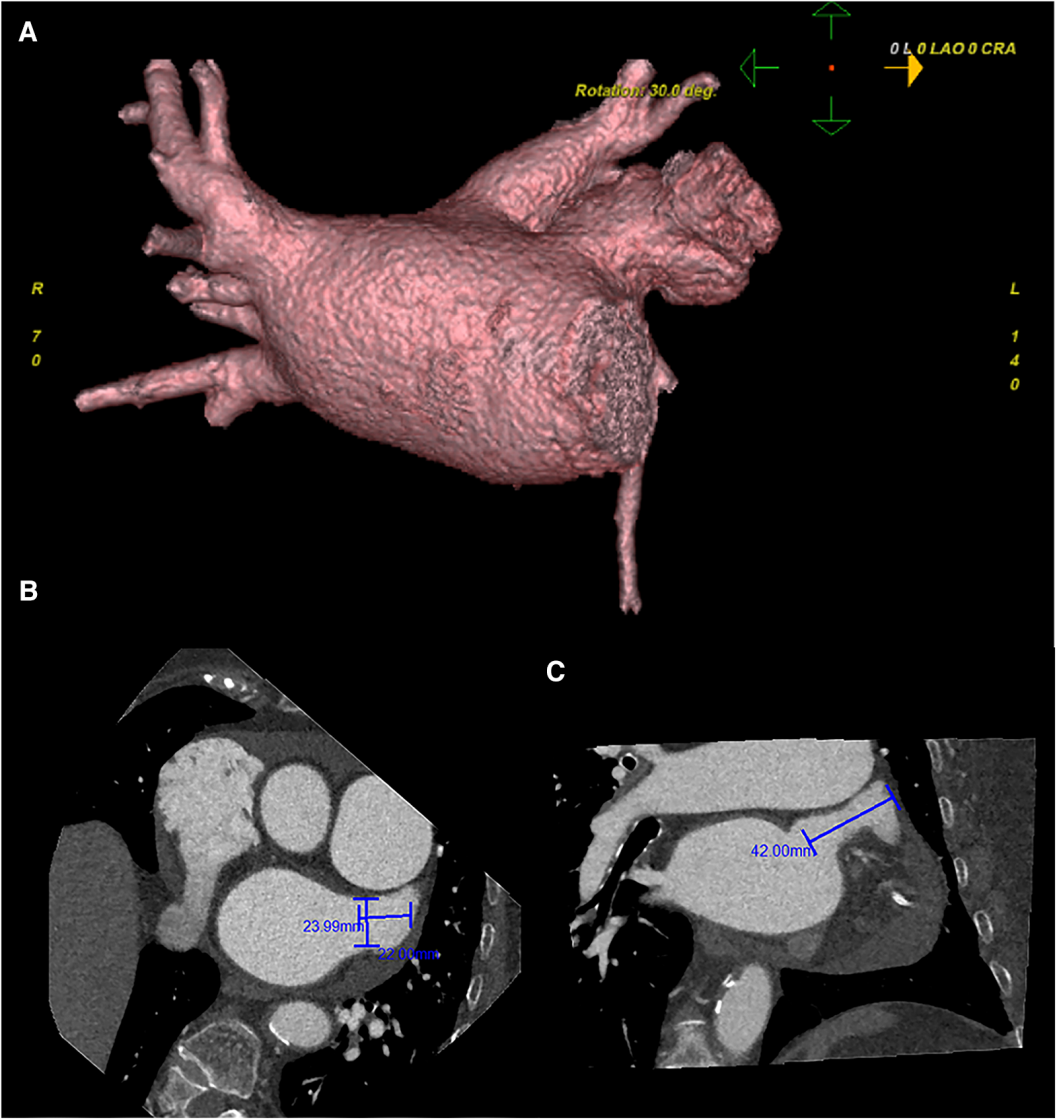
Preoperative cardiac CT angiography reveals a complex left atrial appendage with a large branch, no clear filling defect in the left atrial appendage and left atrium, and no diverticulum and accessory atrial appendage was identified in the left atrium (**A**) the cross-sectional image of the LAA orifice (**B**) the long diameter was about 2.4 cm, the short was about 2.2 cm. Oblique view (**C**) the maximum depth of the left atrial appendage branch was about 4.2 cm. Pericardial effusion.

Preoperative left atrial CT angiography showed that the left atrial appendage was complex with a large branch, while no evident filling defect was observed in both the left atrial appendage and left atrium. No diverticulum or accessory atrial appendage was observed in the left atrium (Figure 1). Laboratory tests on the morning of surgery revealed a hemoglobin count of 117 g/L, a platelet count of 115 × 10^9/L^, and an international normalized ratio (INR) of 0.96.

**Figure 2:**
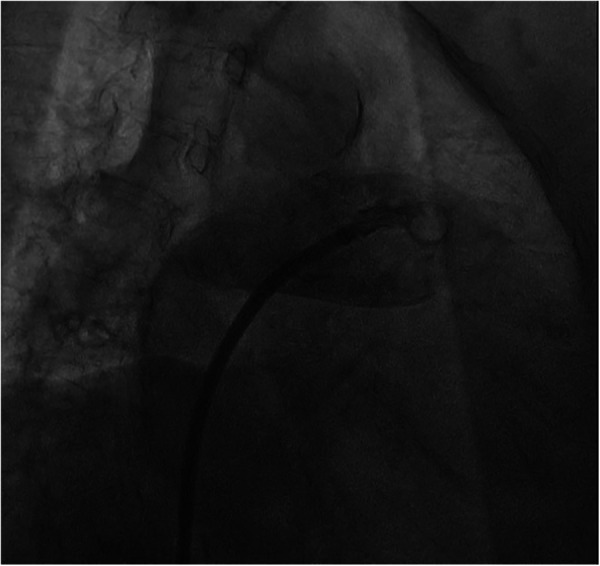
Angiography shows brisk contrast extravasation into the pulmonary artery space.

The procedure was performed under digital subtraction angiography (DSA) guidance, with puncture of the right femoral vein, placement of a 6F sheath, and transseptal puncture using a catheter in the conventional manner. Afterwards, we promptly advanced the transseptal sheath and encountered difficulty in accessing the left superior pulmonary vein with the guidewire, while contrast injection indicated visualization of the pulmonary artery (Figure 2).

Patient experienced a sudden onset of hypotension and tachycardia, intraoperative echocardiography revealed the inadvertent insertion of the delivery tube sheath into the pulmonary artery, accompanied by a pericardial effusion measuring 17 mm. The ultrasound intervention department was promptly contacted for pericardiocentesis and drainage, while norepinephrine and dopamine were administered to augment blood pressure. Following active therapeutic interventions, hemodynamic stability was achieved in the patient.

**Figure 3:**
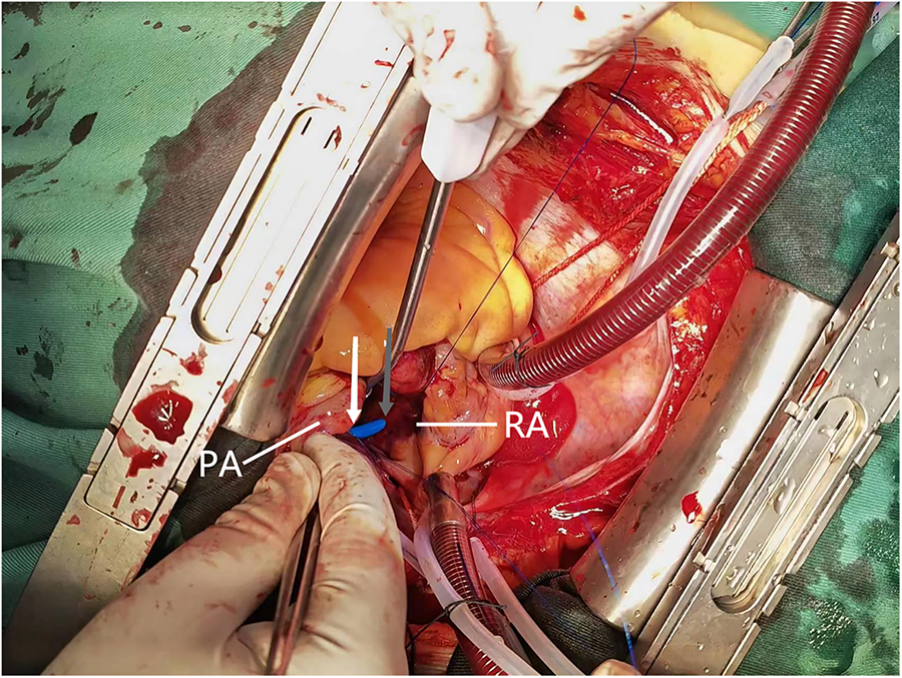
Intraoperative view reveals the pulmonary artery perforation caused by mispiercing of the delivery tube sheath. The grey arrows indicate the exit point of the delivery sheath from the roof of the right atrium, while the white arrows indicate its entry into the main pulmonary artery. PA, pulmonary artery; RA, right atrium.

Cardiac surgery team was consulted to perform surgical exploration in order to identify and suture the tear. A median sternotomy was performed under general anesthesia, and the pericardium was opened to locate the source of bleeding, revealing a subepicardial hematoma in the right atrioventricular groove. Exploration was initiated subsequent to the establishment of cardiopulmonary bypass, revealing that the delivery tube sheath had entered through the superior aspect of the right atrium and traversed through the posterior wall of the right pulmonary artery into the main pulmonary artery (Figure 3). The lacerations of the right atrium and pulmonary artery were repaired using 4-0 prolene sutures, followed by removal of the delivery tube sheath. Subsequently, the left atrial appendage was ligated with double 10-gauge sutures. However, there was still persistent bleeding from the pulmonary artery tear. The main pulmonary artery was dissected under cardiopulmonary bypass, revealing a right posterior wall tear upon exploration from within the pulmonary artery. The gasket from the pulmonary artery suture rupture is reinforced with a 4-0 prolene line. The pulmonary artery incision is sutured with a 4-0 prolene line. Hemostasis is achieved by reinforcing the ruptured area of the pulmonary artery with a 4-0 prolene line. After thorough examination of the right atrial wall and pulmonary artery, the cardiopulmonary bypass machine was ceased upon confirming absence of any bleeding. Protamine was administered to neutralize the effects of heparin, followed by placement of pericardial and mediastinal drainage tubes post achieving hemostasis. The procedure was subsequently concluded in a conventional manner, with an intraoperative blood loss of approximately 600 ml.

Patient was transferred back to the intensive care unit for a day of close monitoring. On the second postoperative day, she returned to the cardiovascular surgery department and initiated anticoagulant therapy with low molecular weight heparin (LVMH). Two days later, she was transferred back to the cardiology department with a gradual reduction in pericardial and mediastinal drainage and discharged on postoperative day 25. Dual antiplatelet therapy with Indobufen and Clopidogrel bisulfate was regularly applied outside the hospital. After seven days, the patient returned to the outpatient clinic exhibiting no subjective symptoms and normalization of N-terminal pro-B-type natriuretic peptide levels (Table 1).

**Table 1:** Timeline of events related to the patient's current procedure.

Time	Event
Baseline	Coronary atherosclerotic heart disease, thrombosis of the left upper limb artery, congestive heart failure, hypertension, ischemic cerebrovascular disease, endometrial cancer, contraindication for long-term anticoagulation.
Day 0	LAAC was performed under the guidance of DSA, angiography after atrial septal puncture showed pulmonary artery visualization. Urgent echocardiography revealed that the delivery tube sheath had inadvertently entered the pulmonary artery, triggering a pericardial effusion. Pericardiocentesis and drainage were performed and vasopressor drugs were given to maintain hemodynamic stability Surgical exploration revealed the inadvertent puncture of the superior aspect of the right atrium by the birth sheath, which subsequently extended into the right posterior and main pulmonary arteries. The right atrium and pulmonary artery were repaired and sutured, while concurrently ligating the left atrial appendage. Pericardial and mediastinal drains were inserted for postoperative management.
Day 1	Treatment was continued in the ward, with the gradual reduction of pericardial effusion.
Day 25	Hospital discharge (Indobufen and Clopidogrel).
Day 32	Asymptomatic.

## Discussion

Left atrial appendage closure has proven to be an effective alternative treatment to long-term oral anticoagulation for stroke prevention in patients with nonvalvular atrial fibrillation (3, 4). The advancement of interventional therapy relying on left heart access has propelled transseptal puncture to attain a highly standardized procedure (1, 2). Although transseptal puncture has an excellent safety profile, complications including cardiac perforation and tamponade, persistent atrial septal defect, thrombosis and embolism cannot be avoided (1, 2). Cardiac tamponade is a relatively common and highly lethal complication that can occur during transseptal puncture, with an overall incidence of approximately 1% (7). The underlying cause is perforation due to inadvertent advancement of the needle or sheath into the free atrial wall or adjacent vessels during transseptal puncture.

The operation of DSA-guided TSP is relatively straightforward and safe, but anatomical abnormalities may obscure the exact position of the needle or even mislead to the wrong puncture point. Therefore, it is crucial to evaluate the patient's left atrial appendage condition and anatomical morphology prior to surgery. Transesophageal echocardiography (TEE) enables clear determination of LAA thrombus presence, as well as measurement of basal diameter and depth, providing a foundation for the operator in selecting the appropriate occluder model. Cardiac CT angiography (CCTA)enables objective and accurate assessment of the left atrial appendage's shape, lobulation, and orifice parameters in the lateral aspect, providing a more comprehensive basis for determining the need for left atrial appendage occlusion. The utilization of these two methods enables the execution of multiplanar imaging and three-dimensional reconstruction, thereby establishing a solid foundation for medical professionals to formulate treatment plans and assess potential risks. Additionally, cardiac magnetic resonance imaging (CMR) can also offer high-quality multiplanar images. For patients with contraindications or intolerance to TEE, intracardiac echocardiography (ICE) can serve as an alternative method for obtaining high-quality multiplanar images (8).

**Figure 4:**
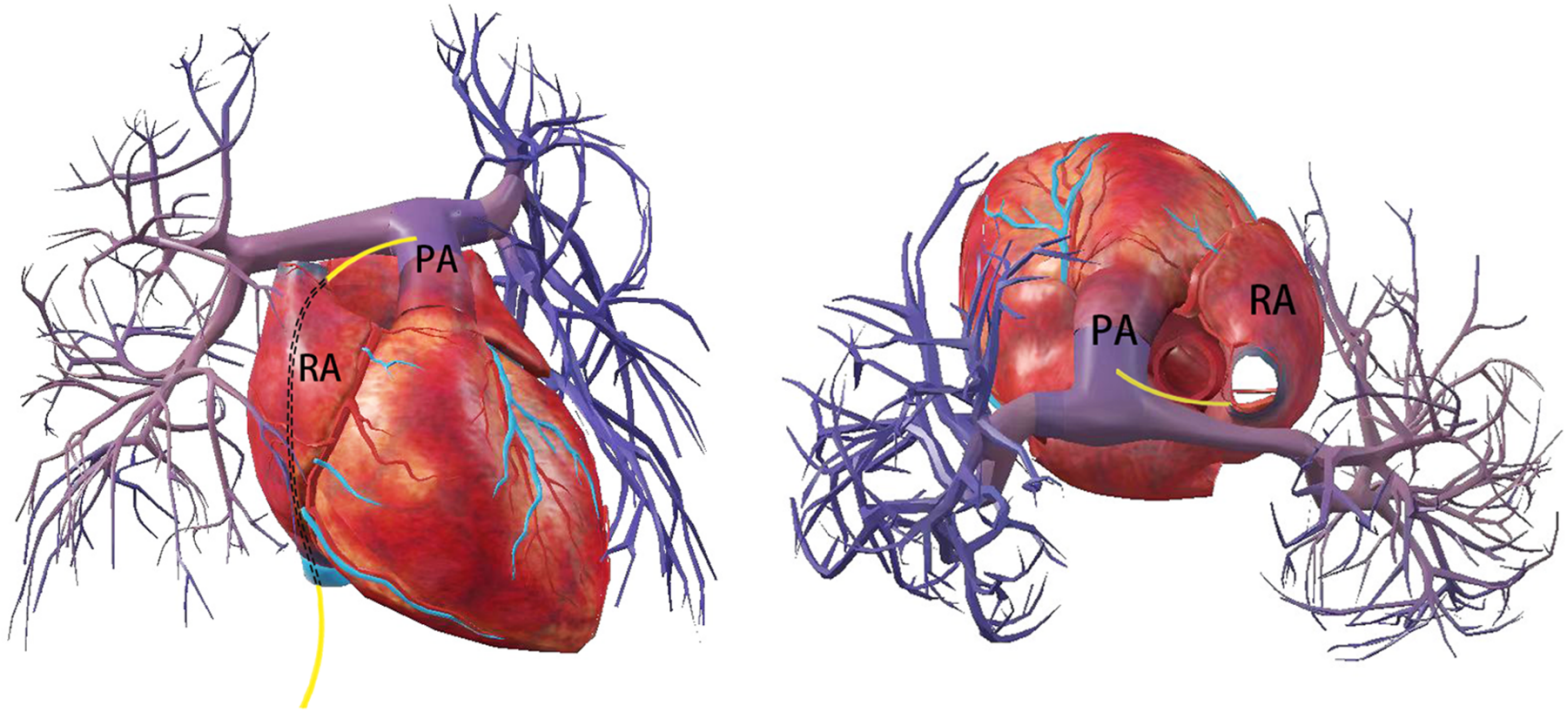
Schematic indicates that the delivery tube sheath enters the right atrium through the inferior vena cava, exits from the roof of the right atrium and punctures the main pulmonary artery leading to perforation.

Previous cases have shown that pulmonary artery injury is the most common type of collateral vessel injury during left atrial appendage occlusion (9) which mostly occurs during LAA occluder implantation. The stabilization hook of ACP/Aamulet, the metal strut of Watchma (9, 10) or the LAmbre device (11) can result in pulmonary artery perforation. It often manifests as cardiac tamponade with hemodynamic failure, which can lead to death in severe cases (9). In the present case, we describe for the first time a pulmonary artery perforation that occurred during transseptal puncture. The mechanism of injury in this case may be that the atrial septal puncture point was too high, the delivery sheath accidentally penetrated the top of the right atrium and moved forward along the transverse pericardial sinus, leading to pulmonary artery perforation, which subsequently triggered cardiac tamponade (Figure 4).

Several cases have been reported in which a percutaneous occluder was utilized to block aortic or cardiac perforations (12, 7) during transseptal puncture. However, as cardiac tamponade can be life-threatening, emergency pericardiocentesis or surgical intervention appears to be the most promising therapeutic option to close the atrial leak (7). For hemodynamically unstable acute pericardial tamponade, ultrasound-guided pericardiocentesis and drainage should be performed first (1, 2). The accumulated blood can be withdrawn and observed if the bleeding volume is minimal and the bleeding rate is slow. The pigtail catheter can be inserted for continuous pericardial drainage in cases of significant and rapid bleeding. To avoid the fatal consequences of active bleeding, it is imperative to promptly request cardiac surgical intervention while ensuring continuous drainage. Fenestrated drainage and cardiac rupture sutures are crucial and effective treatments that should be performed urgently.

Combined with previous literature recommendations, when we encountered complications of pulmonary artery perforation, emergency cardiac decompression was performed immediately, followed by surgical suturing of the ruptured artery and ligation of the left atrial appendage in order to avoid active bleeding. Postoperative follow-up showed that the patient recovered well, which confirmed that surgical repair was an effective treatment.

## Conclusion

Pulmonary artery perforation is a rare but critical complication associated with left atrial appendage closure, which may occur during transseptal puncture. Clinicians should maintain vigilance for uncommon complications and actively enhance preoperative assessment in order to decrease the occurrence of complications. In the event of such complications, emergency surgical exploration and ligation of the left atrial appendage represent a feasible treatment option.

## Patient perspective

“Although a rare complication occurred and the left atrial appendage closure was not successfully completed, I was fortunate to undergo timely surgical intervention, ensuring my safety. I express my gratitude to the medical staff for their exceptional treatment. I hope that my experience can serve as a valuable reference for similar cases”.

## Data Availability

The original contributions presented in the study are included in the article/Supplementary Material, further inquiries can be directed to the corresponding authors.
